# Star fruit toxicity: a cause of both acute kidney injury and chronic kidney disease: a report of two cases

**DOI:** 10.1186/s13104-015-1640-8

**Published:** 2015-12-17

**Authors:** R. A. Abeysekera, S. Wijetunge, N. Nanayakkara, A. W. M. Wazil, N. V. I. Ratnatunga, T. Jayalath, A. Medagama

**Affiliations:** Nephrology and Transplantation Unit, Teaching Hospital, Kandy, Sri Lanka; Department of Pathology, Faculty of Medicine, University of Peradeniya, Kandy, Sri Lanka; Department of Medicine, Faculty of Medicine, University of Peradeniya, Kandy, Sri Lanka

**Keywords:** Star fruit, Nephrotoxicity, Oxalate nephropathy

## Abstract

**Background:**

Star fruit (*Averrhoa carambola*) is commonly consumed as a herbal remedy for various ailments in tropical countries. However, the dangers associated with consumption of star fruit are not commonly known. Although star fruit induced oxalate nephrotoxicity in those with existing renal impairment is well documented, reports on its effect on those with normal renal function are infrequent. We report two unique clinical presentation patterns of star fruit nephrotoxicity following consumption of the fruit as a remedy for diabetes mellitus—the first, in a patient with normal renal function and the second case which we believe is the first reported case of chronic kidney disease (CKD) due to prolonged and excessive consumption of star fruits.

**Case presentation:**

The first patient is a 56-year-old female diabetic patient who had normal renal function prior to developing acute kidney injury (AKI) after consuming large amount of star fruit juice at once. The second patient, a 60-year-old male, also diabetic presented with acute on chronic renal failure following ingestion of a significant number of star fruits in a short duration with a background history of regular star fruit consumption over the past 2–3 years. Both had histologically confirmed oxalate induced renal injury. The former had histological features of acute tubulo-interstitial disease whilst the latter had acute-on-chronic interstitial disease; neither had histological evidence of diabetic nephropathy. Both recovered over 2 weeks without the need for haemodialysis.

**Conclusion:**

These cases illustrate the importance of obtaining the patient’s detailed history with respect to ingestion of herbs, traditional medication and health foods such as star fruits especially in AKI or CKD of unknown cause.

**Electronic supplementary material:**

The online version of this article (doi:10.1186/s13104-015-1640-8) contains supplementary material, which is available to authorized users.

## Background

Star fruit (*Averrhoa carambola*), locally known as “Kamaranka”, is believed to have originated from Ceylon and Moluccas and is now a popular fruit in tropical and subtropical countries [1]. This plant has both nutritional as well as medicinal uses [2]. In Sri Lanka, traditional use of the plant is common for conditions such as diabetes mellitus due to its hypoglycaemic effects [3]. Despite the frequency of use, the dangers of consumption of star fruit are not known to many. Star fruit induced nephrotoxicity, manifesting as acute kidney injury (AKI) and neurological impairment in individuals with previous renal impairment is well documented [4, 5]. However, AKI developing in individuals with normal renal function is rare [6]. We report two unique cases of star fruit nephrotoxicity whereby the first patient had normal renal function prior to developing AKI. We believe the second patient is the first reported case of chronic kidney disease (CKD) due to prolonged consumption of star fruit.

## Case presentation

### Case report 1

A 56-year-old female with well controlled diabetes mellitus for 12 years and no history of diabetic nephropathy presented with generalized weakness and lethargy for 12 days duration.

**Fig. 1:**
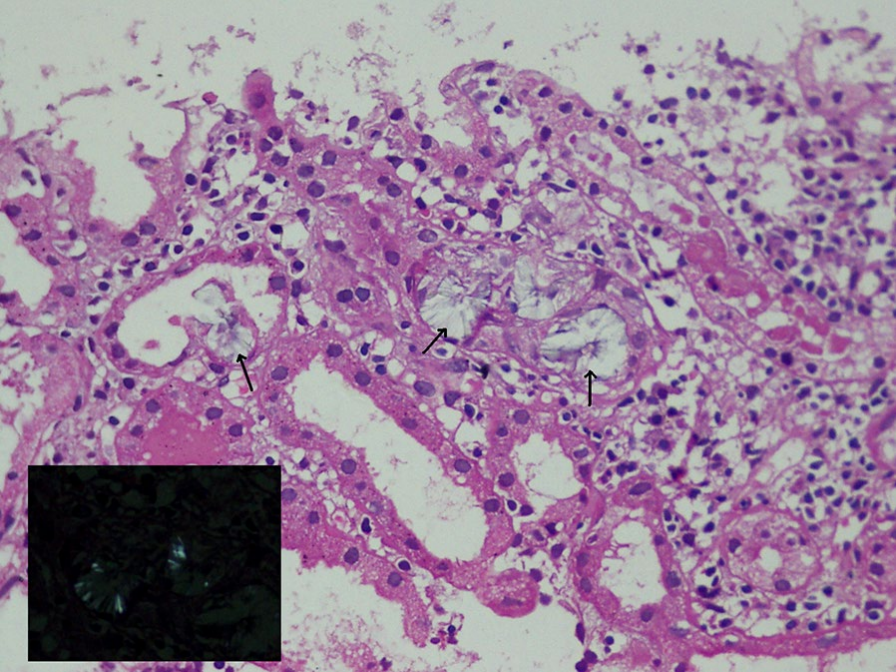
Histology (haematoxylin and eosin stain) of the renal biopsy of case 1 showing acute oxalate induced tubulo-interstitial nephritis. Tubules are blocked by oxalate crystals (*black arrows*). The crystals show polarization with polarizing light (*inset*). The interstitium shows moderate numbers of lymphocytes

12 days prior to presentation, whilst on a pilgrimage, she had consumed 200 ml of concentrated juice of six star fruits as a herbal remedy for diabetes mellitus. 5 h following the ingestion she had developed bouts of severe nausea, vomiting and abdominal discomfort which settled the following morning. Over the next few days she had felt weak and tired with loss of appetite and noticed a mild reduction in urine output. Her diabetes was well controlled on Insulin. Apart from that she was not on any other medication or vitamin C. She had no prior history of urinary tract stones or a family history of kidney disease. On presentation her clinical examination was unremarkable with no neurological signs and a blood pressure of 138/80 mmHg. Investigations revealed a serum creatinine which had risen from her baseline value of 80 µmol/L and eGFR of 68.4 ml/min/1.73 m^2^ from 1 month before to 290 µmol/L with urine full report showing trace amounts of protein and 20–25 pus cells/hpf. Urine culture did not reveal any bacterial growth. Ultrasound scan of kidneys revealed normal kidneys with normal echogenicity with preserved cortico-medullary demarcation. Renal biopsy confirmed the presence of oxalate induced acute tubulo-interstitial nephritis of moderate to severe degree characterized by the presence of tubular obstruction and associated damage by many oxalate crystals which polarized with polarizing light (Fig. 1). The interstitium was oedematous and had a moderate infiltration of lymphocytes. Evidence of CKD such as interstitial fibrosis, tubular atrophy and glomerulosclerosis were not present. Glomerular features of diabetic nephropathy were not present. A short course of oral prednisolone was given due to the presence of interstitial nephritis and her serum creatinine normalized to previous value of 85 µmol/L with an eGFR of 68.4 ml/min/1.73 m^2^ within 3 weeks and she did not require haemodialysis.

### Case report 2

**Fig. 2:**
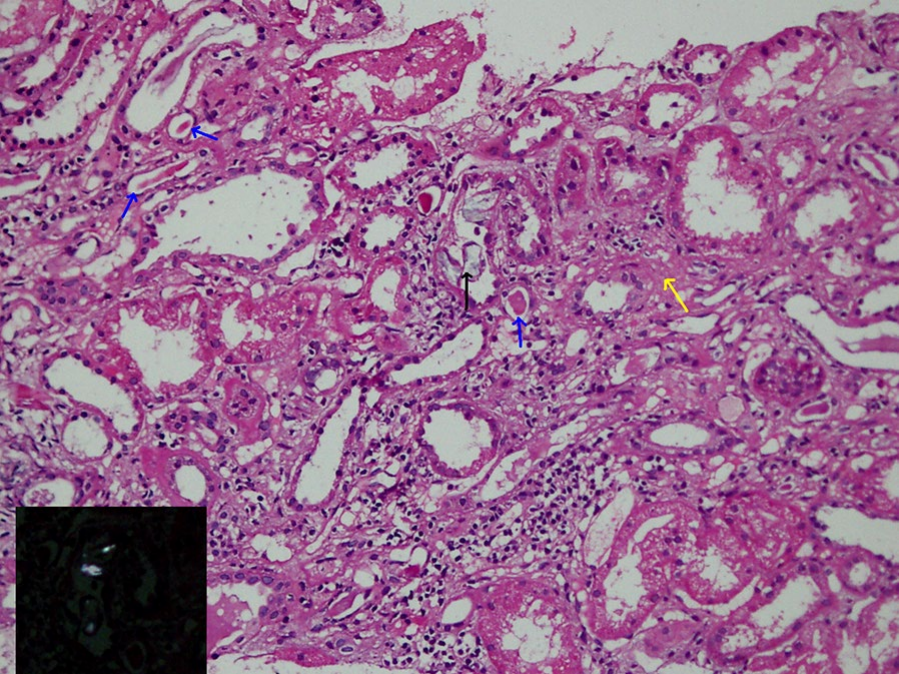
Histology (haematoxylin and eosin stain) of renal biopsy of case 2 showing acute on chronic oxalate induced tubulo-interstitial nephritis. One tubule contains oxalate crystals (*black arrow*) which polarize with polarizing light (*inset*). Evidence of chronic interstitial nephritis, i.e. intersititial fibrosis (*yellow arrow*) and tubular atrophy (*blue arrow*) are present. The interstitium contains a moderate numbers of lymphocytes

A 60-year-old male patient who has had well controlled diabetes mellitus for 5 years presented with rapidly rising serum creatinine from his baseline value of 133 µmol/L and an eGFR of 50.6 ml/min/1.73 m^2^ to 312 µmol/L within 1 week. There was a trace of protein in his urine. However, physical examination revealed no evidence of diabetic retinopathy nor neuropathy. 1 week prior to presentation he had consumed four star fruits over the course of 4 days. Following that, he had felt ill with nausea and weakness. Medication history revealed that he was on Tolbutamide for his diabetes and Simvastatin for his dyslipidemia. Apart from that there was no other history of drug consumption, vitamin C use, herbal medication or substance abuse. He had no family history of renal stone disease. On examination he was not dehydrated, his blood pressure was 146/88 mmHg. Ultrasound scan of kidneys showed normal kidneys with normal echogenicity with preserved cortico-medullary demarcation. Renal biopsy revealed the presence of oxalate induced tubulo-interstitial nephritis of moderate degree. There were several tubules obstructed by oxalate crystals which polarized with polarizing light and some crystals were calcified (Fig. 2). The interstitium contained a moderate lymphocytic infiltration. There were evidence of chronic kidney injury characterized by presence of interstitial fibrosis and tubular atrophy of moderate degree. Glomerular changes of diabetic nephropathy were not present. Accordingly, the overall histological appearance was consistent with those of acute on chronic oxalate induced nephropathy. Enquiring retrospectively, it was found that this patient had been consuming 5–6 star fruits every month for 2–3 years. However, this was the first time such a number was consumed over a short period of time. He had a good urine output and did not require haemodialysis. After 2 weeks his serum creatinine returned to his baseline of 130 µmol/L and an eGFR of 51.91 ml/min/1.73 m^2^.

## Discussion

Star fruit is a rich source of oxalates. Nephrotoxicity leading to AKI is a result of both tubular obstruction by calcium oxalate crystals as well as apoptosis of the renal tubular epithelial cells [7]. Risk factors for developing toxicity have been described as previously impaired renal function, ingestion of large amount of fruits or ingestion on an empty stomach [8]. The symptoms are known to occur within hours of ingestion with the most well described being hiccups, nausea, vomiting and neuropsychiatric manifestations such as insomnia or seizures. Demonstration of oxalate crystals obstructing the tubules is diagnostic of star fruit induced nephropathy. Some patients with AKI can be managed without renal replacement therapy, but the majority require early renal replacement therapy in the form of haemodialysis or haemoperfusion [6, 9]. Peritoneal dialysis is ineffective especially in the presence of neurological manifestations [10].

Of the cases we report here, the first patient had consumed a large volume of star fruit juice at one sitting. It has been reported that even a volume of 25 ml would be adequate to cause nephrotoxicity [10]. In the literature, most reported cases with oxalate induced nephrotoxicity had underlying renal dysfunction due to other causes and developed acute-on-chronic renal failure which has been described as potentially lethal [11]. This case is unique in that she developed AKI with underlying normal renal function. There were only two other similar cases reported in literature [6, 8].

The second patient developed acute on CKD due to star fruit toxicity with prior mildly deranged renal functions. In the absence of clinical evidence of diabetic retinopathy and histological evidence of diabetic nephropathy, the pre-existing renal dysfunction cannot be attributed to diabetes mellitus. Histology of the renal biopsy also confirmed presence of chronic tubuo-interstitial renal disease. Detailed history did not reveal any other aetiology except the fact that he had prolonged consumption of moderate volumes of star fruit with recent ingestion of a large quantity over the course of 4 days. Accordingly, it could be postulated that the prolong consumption of star fruit had led to recurrent occult renal damage resulting in chronic interstitial nephritis and CKD. We believe this is the first reported case of its kind in the literature. It has been shown that calcium oxalate is one of the most reactive crystals and can evoke an inflammatory response leading to interstitial fibrosis, loss of nephrons, and eventually to chronic renal failure which further validates this point [12].

## Conclusion

It is imperative that a history with respect to ingestion of foods such as star fruits is inquired especially in patients with AKI or CKD of unknown cause. Moreover, medical professionals should advise patients with CKD or those on dialysis to avoid eating star fruit. Even individuals with normal renal function should be careful not to ingest star fruits in excessive quantities since the second report shows that even regular moderate ingestion is harmful.

## Consent

Written informed consent was obtained from both patients for publication of this case report and accompanying images (Additional file 1).

## Additional files

10.1186/s13104-015-1640-8 CARE Checklist (2013) of information to include when writing a case report.

## Abbreviations

AKI: acute kidney injury
CKD: chronic kidney disease
